# Gamma Ray Spectrometric Analysis of Sand Samples from Selected Beaches along Kenyan Coastline

**DOI:** 10.1155/2021/6621645

**Published:** 2021-02-23

**Authors:** Willis Otieno Gor Odongo, Nadir Hashim, Margaret Wairimu Chege

**Affiliations:** ^1^Department of Physics, Kisii University, P.O. Box 408, Kisii, Kenya; ^2^Department of Physics, Kenyatta University, P.O. Box 43844, Nairobi, Kenya

## Abstract

In this study, the activity concentration levels of ^238^U, ^232^Th, and ^40^K in sand samples collected from Shanzu, Nyali, Kenyatta, Tiwi, Shelly, and Diani beaches selected along the Kenyan coastline were determined using a gamma ray spectrometer with a NaI(Tl) detector. The average activity concentrations of ^238^U, ^232^Th, and ^40^K in sand samples were analyzed as 87 ± 4, 98 ± 4, and 1254 ± 62 Bq/kg, respectively. Also, radium equivalent (Ra_eq_) activity and internal (*H*_in_) and external (*H*_ex_) hazard index were calculated to assess the radiological hazards associated with the use of sand samples as building materials. The average values of Ra_eq_, *H*_in_, and *H*_ex_ were found as 327 ± 16 Bq/kg, 0.98, and 0.72, respectively. The average values of outdoor and indoor annual effective dose rates were estimated as of 0.23 and 0.63 mSv/y, respectively, which are below maximum recommended limit of 1 mSv/y. Generally, these results indicate no significant radiological health hazards for the studied beaches.

## 1. Introduction

Natural radionuclides originated from nucleo-synthesis process. Radionuclides such as ^40^K, ^238^U, and ^232^Th which have half-lives comparable to the age of the Earth are to date present in geological materials. They occur in varying amounts in rock and soil as characterized by geology of a place [1]. They contribute the largest fraction of natural radiation exposure to the general public [2]. Sands on the beach are weathering impervious residuals of geological processes. They may have gotten to the coastline either through erosion from mainland or were deposited through waves and currents actions. Waves normally backwash the lighter sand grains while the heavier ones remain on the beach, which contains minerals like zircon, ilmenite, garnet, and monazite [3] which are associated with the presence of naturally occurring radionuclides.

Research on levels of radionuclide concentration and the associated radiological hazards in soils, water, air, and sediments on the Kenyan coastal region has been done [4–7]. Radioactivity levels from natural radionuclides on Kenyan beaches are not known despite the fact that there is a need to determine the reference levels more so for areas with higher risk of radioactive materials exposure [8]. Beach sands may be exposed to these radioactive materials through wave action to traces of oil spillages and dumped radioactive wastes in the Indian Ocean waters, all of which have high association with these radionuclides. People spend time relaxing and sun-bathing on these beaches oblivious of the possible danger of exposure to naturally occurring radiation. The beach sand is also used for construction of houses by the coastal communities and therefore the need for the determination of risks associated with the radiation exposure from such houses.

This study focused on the coastline near the port city of Mombasa. The coastline is divided with respect to the city into two zones: south coast and north coast. Six popular beaches, three from each zone, were considered: Diani, Tiwi, and Shelly on the south coast and Shanzu, Kenyatta, and Nyali on the north coast. Most south coast beaches are of sheltered type with relatively coarse sand grains compared to the ones on the north coast which are majorly exposed type with more fine-grained sand particles [9].

## 2. Materials and Methods

### 2.1. Study Area

In south coast, Diani Beach is 30 kilometers south of Mombasa in Kwale County and is the most popular, majorly favored by foreign tourists. Tiwi is approximately 21 kilometers south of Mombasa also in Kwale County, majorly composed of white sands and an outstretched large reef resulting in shallow waters. Shelly Beach is the closest to Mombasa and is favored by local residents due to easy accessibility.

In north coast, Nyali Beach is closest to Mombasa known for its many high-class hotels and long white sands stretches. It is part of the Mombasa Marine Reserve. Kenyatta Beach is one of the few public beaches frequented by domestic tourists. It is located 500 meters from Mombasa-Malindi road and the most popular on the North. Shanzu Beach is located further north. It is in the vicinity of a number of popular hotels, bars, and restaurants.

### 2.2. Sample Collection and Preparation

**Figure 1:**
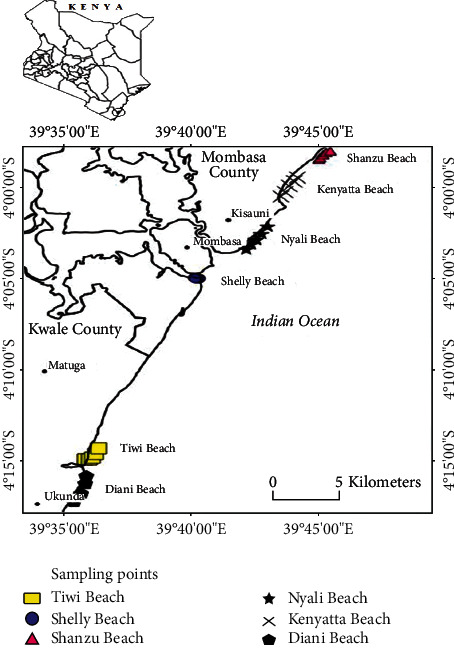
A map of the coastline of Kenya showing the sampling points in this work. The sampling points are indicated by the colored dots.

Thirty sand samples, 5 from each of the 6 beaches, were collected approximately at a separation distance of 0.50 km to 1.50 km apart along the accessible points on the beaches between Diani beach on south coast and Shanzu Beach on north coast as shown in Figure 1. The sand samples were collected from an area of 0.25 m by 0.25 m; the area was first cleared of debris and then sand was scooped using a shovel to an approximate depth of 0.10 m.

The exact position for each sand sample was determined using hand-held Garmin GPS and then recorded. Each sand sample was then kept in a plastic container and labelled based on the location where it was obtained. The sand samples were sieved through the sieves of different diameters depending on the grain size, a process known as sieve test. The sand samples were dried in an oven at a temperature of 110°C for 24 hours to remove the water content. 400 g mass of each of the sand samples was placed in hermetically sealed polythene bags to prevent radon from leaking. The labelled sample bottles and the reference materials were stored for about 4 weeks to allow ^226^Ra and ^232^Th atoms to attain secular equilibrium with their respective short-lived progeny.

Thallium activated sodium iodide [NaI(Tl)] detector was used to measure the activity concentration of the radionuclides in each of the sand samples for 30,000 seconds to increase precision of radiometric measurements.

## 3. Experimental Techniques

### 3.1. Gamma Ray Spectrometry

Thallium activated sodium iodide [NaI(Tl)] gamma ray spectrometer was used in the detection, identification, and measurement of radionuclides in the samples. The spectrometry system has a 76 mm × 76 mm single crystal of thallium activated sodium iodide detector, an Oxford PCAP multichannel analyzer (MCA) which is a PC-based plug-in PCI card consisting of an 80 MHz Wilkinson Analogue-to-Digital Converter.

Gamma rays from sand sample strike the NaI(Tl) crystal emitting photons which ejects electrons from the photocathode which are multiplied in the photomultiplier tube. Preamplifier attached to the detector collects the charges produced by the photomultiplier tube and produces a voltage proportional to the input charge (pulses). The amplifier shapes the voltage pulses and increases their sizes. The multichannel analyzer digitizes the voltage pulse and displays the results through a personal computer.

Energy calibration of the NaI(Tl) detector was done using Ceasium-137 at energy peak of 662 keV and cobalt-60 at energy peaks of 1170 keV and 1330 keV.

Efficiency calibration of the detector was done using IAEA standard certified reference materials: RGU-1, RGTh-1, and RGK-1 of energies 4940, 3250, and 14,000 Bq/kg, respectively; the samples were counted for 30,000 seconds.

The energy resolution of the detector was determined by measuring a standard source of ^137^Cs in the detector for 600 s and spectrum obtained at 662 keV energy peak. The energy resolution was then determined as 7.3 ± 1%.

The minimum detectable activity (MDA) was determined by first doing background count using an empty bottle used to hold the samples, for 30,000 s on the NaI (Tl) detector. Using the spectrum of the background counting, the following isotopes and their corresponding energy peaks ^214^Bi (1764.49 keV) and ^208^ Tl (2614.5 keV) and for ^238^U and ^232^Th, respectively, and energy peak of 1460.83 KeV for ^40^K were used to determine their lower limit of detection (LLD) then MDA, respectively.

The background counts were then used for correction of net peak area of gamma rays of the measured standard isotopes. The LLD and MDA were calculated using equations (1) and (2), respectively [10, 11].

(1) $$\begin{array}{c} \text{LLD}=4.66\sigma_{b\;}+3, \end{array}$$

(2) $$\begin{array}{c} \text{MDA}=\;\frac{\text{LLD}}{\varepsilon \text{Yt}}, \end{array}$$

where *σ*_*b* _ is the standard deviation of the background in the region of interest, *ε* is the absolute efficiency of the detector, *Y* is the absolute gamma emission probability of the gamma decay, and *t* is the counting time in seconds.

The MDA for ^40^K, ^232^Th, and ^226^Ra were determined as 1.35 Bq, 0.186 Bq, and 0.386 Bq, respectively.

The background intensity was measured by counting 400 millilitres of distilled water for 30,000 seconds under the same geometry as the sand samples. To obtain the net intensity of each of the sand samples, the background intensity was subtracted from the gross intensity of the sand samples. For ^40^K, the peak energy of 1460 keV was considered; for ^238^U (^214^ Bi), the peak energy of 1765 keV was considered; and for ^232^Th (^208^Tl), the peak energy of 2615 keV was considered for the evaluation of sand sample intensities.

The activity concentration was then determined by the use of comparison method given by[7]

(3) $$\begin{array}{c} \frac{A_{s}M_{s}}{I_{s}}=\;\frac{A_{r}M_{r}}{I_{r}}, \end{array}$$

where *A*_*s*_ is activity of the sand sample, *M*_*s*_ is mass of the sand sample, *I*_*s*_ is peak intensity of the radionuclide in the sand sample, *M*_*r*_ is mass of the reference sample, *A*_*r*_ is activity concentration of the reference sample, and *I*_*r*_ is the peak intensity of the radionuclide in the reference sample.

### 3.2. Radium Equivalent Activity

Radium equivalent is a single value describing gamma output from the three radionuclides (^238^U, ^232^Th, and ^40^K) since their distribution in the environment is not the same. It was calculated using [12]

(4) $$\begin{array}{c} {\text{Ra}}_{\text{eq}}=A_{\text{Ra}}+1.429A_{\text{TH}}+0.0769A_{\text{K}}, \end{array}$$

where *A*_Ra_, *A*_Th_, and *A*_K_ are the activity concentrations of ^226^Ra, ^232^Th, and ^40^K. The 1.429 and 0.0769 are conversion factors for thorium and potassium, respectively.

### 3.3. Radiation Hazard Indices

#### 3.3.1. External Hazard Index (H_ex_)

It was calculated with the assumption that 370 Bq/kg of ^238^U, 259 Bq/kg of ^232^Th, and 4810 Bq/kg of ^40^K produce the same gamma ray dose. It was determined using [13]

(5) $$\begin{array}{c} H_{ex}=\;\frac{A_{Ra}}{370}+\;\frac{A_{Th}}{259}+\;\frac{A_{K}}{4810}. \end{array}$$

The value of this index must be less than unity to keep the radiation risks negligible.

#### 3.3.2. Internal Hazard Index (*H*_in_)

Short-lived radon and its daughter products are internally hazardous to the respiratory organs. The internal exposure to radon and its progenies is referred to as internal hazard index denoted by *H*_in_. It was calculated using [13]

(6) $$\begin{array}{c} H_{in}=\;\frac{A_{Ra}}{185}+\;\frac{A_{Th}}{259}+\;\frac{A_{K}}{4810}. \end{array}$$

### 3.4. Absorbed Dose Rates

#### 3.4.1. Outdoor Gamma Radiation Absorbed Dose Rate

To calculate outdoor absorbed dose rates due to terrestrial gamma rays 1 m above the ground, the following equation was used [14]:

(7) $$\begin{array}{c} D_{o}=0.0417A_{K}+0.462A_{\text{Ra}}+0.604A_{\text{Th}}, \end{array}$$

where 0.0417 nGy/h, 0.462 nGy/h, and 0.604 nGy/h are dose conversion factors coefficients of ^40^K, ^238^U, and ^232^Th, respectively, and its unit is nano gray per hour (nGy/h).

#### 3.4.2. Indoor Gamma Radiation Dose Rate

The indoor absorbed gamma ray radiation dose rate was calculated using [14]

(8) $$\begin{array}{c} D_{i}=0.057A_{K}+0.67A_{\text{Ra}}+0.78A_{\text{Th}}, \end{array}$$

where 0.057, 0.67, and 0.78 are specific dose rates in nGyh^−1^ or Bqkg^−1^ of ^40^K, ^238^U, and ^232^Th, respectively. The parameter values used in calculating these dose rates are based on the model room of dimensions 4 m by 5 m by 2.8 m and were determined by Monte Carlo simulation. The model house is one which the floors and walls are made of concrete that contain the sand from the beaches while the ceiling is made of wooden or plastic materials.

### 3.5. Annual Effective Absorbed Dose Rate

Annual effective absorbed dose rate is the measure of biological effect of radiation on human tissue and it is calculated from the absorbed dose rate using conversion factor and time of occupancy per day for the whole year. Its SI unit is Sievert (Sv).

Both the outdoor and indoor annual effective dose rates in mSvy^−1^ were calculated using [15]

(9) $$\begin{array}{c} E=D\;x\;T\;x\;Q\;x\;10^{-6}, \end{array}$$

where *D* is the absorbed dose rate in nGy/h, *T* is the occupancy time taken as six hours per day outdoors and twelve hours per day indoors, and *Q* is conversion factor of 0.7 Sv/Gy.

## 4. Results and Discussion

### 4.1. Activity Concentration

Table 1 gives the summary of the activity concentration of ^232^Th, ^238^U, and ^40^K. The average activity concentration of ^40^K was 1254 ± 62 Bq/kg with the highest site having a value of 2117 ± 105 Bq/kg and the lowest site having 653 ± 32 Bq/kg. ^238^U had an average value of 87 ± 4 Bq/kg with values ranging between 118 ± 5 Bq/kg and 43 ± 2 Bq/kg. ^232^Th had a mean value of 98 ± 4 Bq/kg with values ranging between 127 ± 6 Bq/kg and 42 ± 2 Bq/kg.

The variation in the measured values was analyzed using a one-way ANOVA. No significant variation was found at 0.05 significant level.

The differences in the activity concentrations were possibly due to the following reasons: first, the type of beach, which is either sheltered or exposed. Sheltered beach experiences less erosion as it is protected by the coral reefs or heavy vegetation along the shoreline. Therefore, the sand grains may contain higher concentration of natural radionuclides or have erosion as they are protected by the coral reefs or heavy vegetation along the shoreline. Therefore, the sand grains which may contain higher concentration of natural radionuclides or have traces of oil spillages and other radioactive wastes are not easily washed back to the sea from the beach like in Diani and Tiwi beach leading to higher levels of activity concentration.

Second, the size of sand grains; waves easily wash back the lighter grains leaving behind the heavier ones on the beach, which may be rich in valuable minerals such as zircon, ilmenite, garnet, and monazite which contain the natural radionuclides. Thus, beaches with large sand grains tend to have higher concentration of the radionuclides like Kenyatta Beach.

Activity concentration of ^40^K was generally higher compared to those of ^238^U and ^232^Th. This is because beach sand has high silica content which is attributed to the formation of silicate (SiO_2_) during cooling and solidification of igneous rocks and ^40^K is highly compatible with silica compared to ^232^Th and ^238^U [7].

The concentration of ^238^U and ^232^Th was also found to be higher than the world average; this could be attributed to pollution from oil leakages and radioactive waste damping in the sea which are washed to the beach and are associated with naturally occurring radionuclides.

**Figure 2:**
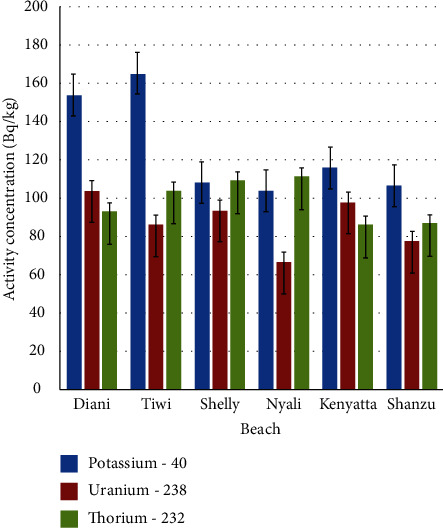
Average activity concentration of ^232^Th, ^238^U, and ^40^K values measured in the sand samples from the six beaches (activity concentration of ^40^K has been reduced by a power of ten for clarity of ^238^U and ^232^Th levels).

The average values of activity concentration in each beach show that Tiwi Beach had the highest average value of activity concentration for ^40^K of 1648.1 ± 82.4 Bq/kg, Diani had highest average of 103.7 ± 5.1 Bq/kg for ^238^U, and Nyali beach had highest average of 111.2 ± 5.5 Bq/kg for ^232^Th as shown in Figure 2.

Generally, south coast beaches (Shelly, Diani, and Tiwi) had higher activity concentration values compared to the north coast beaches (Nyali, Shanzu, and Kenyatta); this could be attributed to the following: first is its proximity to the Mrima Hill which is a high radiation area and is approximately 30 km from Diani and Tiwi Beach as some of the beach sands could be originating from it though erosion agents like River Mwachema which flows to the ocean at Daini Beach; secondly, most of the south coast beaches are of the sheltered type [9]; this means that most radionuclides that are brought to the beach are not easily eroded away; lastly, most beaches in south coast have large sand grains which are associated with higher concentration of radionuclides.

### 4.2. Radium Equivalent

Table 2 gives the values of the radium equivalent from each of the thirty samples and the average in each beach.

From Table 2, radium equivalent had a mean of 327 ± 16 Bq/kg with its values ranging from a maximum value of 455 ± 23 Bq/kg to a minimum value of 220 ± 11 Bq/kg.

**Figure 3:**
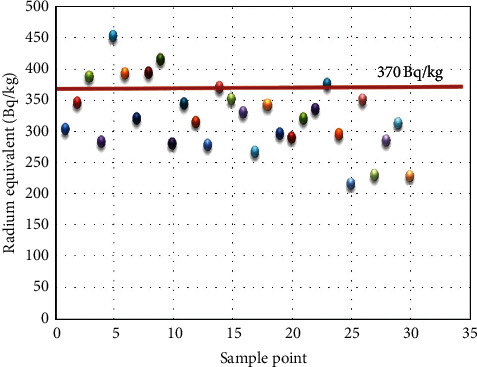
Radium equivalent values in the samples analyzed in this work. The solid line indicates an average safety limit value of 370 Bq/kg [16].

The scatter plot of radium equivalent shown in Figure 3 indicates that only seven sample points of the studied points recorded values above the recommended limit of 370 Bq/kg.

### 4.3. Hazard Indices

The values of external and internal hazard indices in the sand samples are tabulated in Table 3 with average values of 0.72 ± 0.03 and 0.98 ± 0.04, respectively, which are both less than unity and thus safe according to [16].

From Table 3, thirteen samples had internal hazard index above unity limit while for external hazard index only one sample point was above this limit. All averages for external hazard index per beach were below the limit of one.

### 4.4. Absorbed Dose Rate and Annual Effective Dose Rates

The indoor absorbed dose rate varied from between 143 ± 7 nGy/h and 292 ± 14 nGy/h with a mean of 207 ± 8 nGy/h which is above the world average of 84 nGy/h. Outdoor absorbed dose rate varied between 102 ± 5 nGy/h and 211 ± 10 nGy/h with a mean of.150 ± 7 nGy/h which is higher than the world average of 54 nGy/h [17].

The annual effective dose rates are shown in Table 4. The values of indoor annual effective dose rate varied between 0.43 ± 0.02 mSv/y and 0.89 ± 0.04 mSv/y with a mean of 0.63 ± 0.03 mSv/y. Outdoor annual effective dose rate ranged between 0.16 ± 0.01 mSv/y and 0.32 ± 0.02 mSv/y with a mean of 0.23 ± 0.01 mSv/y. The average values for both indoor and outdoor annual effective dose rate were higher than world averages of 0.41 msv/y and 0.07 mSv/y, respectively, but less than the safety value of one in all the beaches [18].

The results obtained in this work have been compared with results reported in other beaches in Kenya and others around the world in Table 5 [17, 19–24]. There is no great variation of concentration of these radionuclides except for Tamil Nadu, India, which had very high values.

## 5. Conclusion

The activity concentration levels for ^238^U, ^40^K, and ^232^Th along the selected coastline beaches have been measured using the gamma ray spectrometer system. The radiological parameters of these beach sands have been determined alongside their radiological effects on human beings.

The variation in the activity concentrations is attributed to the following: firstly, the differences in types of the beaches which are either sheltered or exposed with the sheltered type tending to have higher concentration; secondly, the variation in the size of sand grains as waves wash back the lighter grains leaving behind the heavier ones on the beach, which may be rich in valuable minerals such as zircon, ilmenite, garnet, and monazite which contain the natural radionuclides, thus higher concentration like on Kenyatta Beach; thirdly, the level of pollution from oil spillages in the sea waters reaching the beach which mostly affects concentration of ^238^U and ^232^Th like on Nyali and Shelly Beaches, only that Nyali has finer sand which allows easier backwash of the pollutants.

Radium equivalent had an average value below the recommended limit of 370 Bq/kg as only seven sample points recorded values above this limit with six of them from the south coast.

The average values for external and internal hazard indices were below the recommended limit of one making the beach sand safe according to [16] even though thirteen samples had an internal hazard index above unity limit, nine of which are from the south coast while only one sample point was above this limit for the external hazard index.

The indoor and outdoor absorbed dose rates both had average values above the world average of 84 nGy/h and 54 nGy/h, respectively [18].

The indoor and outdoor annual effective dose rates had mean values higher than world averages of 0.41 mSv/y and 0.07 mSv/y, respectively. However, the total annual effective dose rate was less than the safety limit of 1 mSv/y [18].

## Figures and Tables

**Table 1:** Activity concentration of^40^K, ^238^U, and^232^Th in the sand samples in this work.

Site	40K (Bq/kg)	238U (Bq/kg)	^232^Th (Bq/kg)
Diani-1	1621 ± 81	103 ± 5	53 ± 2
Diani-2	1246 ± 62	105 ± 5	102 ± 5
Diani-3	1609 ± 80	100 ± 5	114 ± 5
Diani-4	1101 ± 55	90 ± 4	76 ± 3
Diani-5	2101 ± 105	118 ± 5	119 ± 5
Tiwi-1	1936 ± 96	72 ± 3	114 ± 5
Tiwi-2	1294 ± 64	73 ± 3	104 ± 5
Tiwi-3	2117 ± 105	86 ± 4	110 ± 5
Tiwi-4	1803 ± 90	95 ± 4	126 ± 6
Tiwi-5	1089 ± 54	107 ± 5	63 ± 3
Shelly-1	1064 ± 53	90 ± 4	121 ± 6
Shelly-2	1319 ± 65	69 ± 3	101 ± 5
Shelly-3	653 ± 32	95 ± 4	95 ± 4
Shelly-4	1331 ± 66	115 ± 5	108 ± 5
Shelly-5	1033 ± 51	94 ± 4	125 ± 6
Nyali-1	1190 ± 59	57 ± 2	128 ± 6
Nyali-2	1263 ± 63	43 ± 2	89 ± 4
Nyali-3	1066 ± 53	87 ± 4	122 ± 6
Nyali-4	774 ± 38	72 ± 3	117 ± 5
Nyali-5	907 ± 45	78 ± 3	101 ± 5
Kenyatta-1	1379 ± 68	107 ± 5	75 ± 3
Kenyatta-2	1578 ± 78	76 ± 3	96 ± 4
Kenyatta-3	1282 ± 64	108 ± 5	119 ± 5
Kenyatta-4	701 ± 35	104 ± 5	98 ± 4
Kenyatta-5	859 ± 42	90 ± 4	42 ± 2
Shanzu-1	1057 ± 52	89 ± 4	127 ± 6
Shanzu-2	1367 ± 68	45 ± 2	57 ± 2
Shanzu-3	726 ± 36	98 ± 4	93 ± 4
Shanzu-4	1139 ± 56	75 ± 3	106 ± 5
Shanzu-5	1023 ± 51	80 ± 4	49 ± 2
Average	1254 ± 62	87 ± 4	98 ± 4
World average [16]	420	33	45

**Table 2:** The values of radium equivalent for each of the thirty samples and average of each beach in this work.

Site	Ra_eq_ (Bq/kg)
Diani-1	307 ± 15
Diani-2	349 ± 17
Diani-3	391 ± 20
Diani-4	287 ± 14
Diani-5	455 ± 23
Tiwi-1	396 ± 20
Tiwi-2	324 ± 16
Tiwi-3	397 ± 20
Tiwi-4	418 ± 21
Tiwi-5	284 ± 14
Shelly-1	348 ± 17
Shelly-2	318 ± 16
Shelly-3	282 ± 14
Shelly-4	374 ± 19
Shelly-5	355 ± 18
Nyali-1	334 ± 17
Nyali-2	271 ± 14
Nyali-3	346 ± 17
Nyali-4	300 ± 15
Nyali-5	294 ± 15
Kenyatta-1	324 ± 16
Kenyatta-2	339 ± 17
Kenyatta-3	379 ± 19
Kenyatta-4	299 ± 15
Kenyatta-5	234 ± 12
Shanzu-1	354 ± 18
Shanzu-2	220 ± 11
Shanzu-3	289 ± 14
Shanzu-4	316 ± 16
Shanzu-5	232 ± 12
Average	327 ± 16

**Table 3:** The internal and external hazard indices of the sand samples and the averages of each beach.

Site	*H*_ex_	*H*_in_
Diani-1	0.69 ± 0.03	0.99 ± 0.04
Diani-2	0.77 ± 0.03	1.08 ± 0.05
Diani-3	0.87 ± 0.04	1.16 ± 0.05
Diani-4	0.63 ± 0.03	0.90 ± 0.04
Diani-5	1.02 ± 0.05	1.37 ± 0.06
Tiwi-1	0.89 ± 0.04	1.13 ± 0.05
Tiwi-2	0.72 ± 0.03	0.93 ± 0.04
Tiwi-3	0.90 ± 0.04	1.11 ± 0.05
Tiwi-4	0.93 ± 0.04	1.21 ± 0.06
Tiwi-5	0.62 ± 0.03	0.94 ± 0.04
Shelly-1	0.76 ± 0.03	1.03 ± 0.05
Shelly-2	0.71 ± 0.03	0.91 ± 0.04
Shelly-3	0.61 ± 0.03	0.89 ± 0.04
Shelly-4	0.82 ± 0.04	1.16 ± 0.05
Shelly-5	0.77 ± 0.03	1.05 ± 0.05
Nyali-1	0.74 ± 0.03	0.91 ± 0.04
Nyali-2	0.61 ± 0.03	0.73 ± 0.03
Nyali-3	0.76 ± 0.03	1.01 ± 0.05
Nyali-4	0.65 ± 0.03	0.86 ± 0.04
Nyali-5	0.64 ± 0.03	0.87 ± 0.04
Kenyatta-1	0.72 ± 0.03	1.04 ± 0.05
Kenyatta-2	0.76 ± 0.03	0.98 ± 0.04
Kenyatta-3	0.83 ± 0.04	1.15 ± 0.05
Kenyatta-4	0.64 ± 0.03	0.95 ± 0.04
Kenyatta-5	0.54 ± 0.02	0.75 ± 0.03
Shanzu-1	0.77 ± 0.03	1.04 ± 0.05
Shanzu-2	0.48 ± 0.02	0.67 ± 0.03
Shanzu-3	0.62 ± 0.03	0.92 ± 0.04
Shanzu-4	0.70 ± 0.03	0.92 ± 0.04
Shanzu-5	0.51 ± 0.02	0.75 ± 0.03
Average	0.72 ± 0.03	0. 98 ± 0.04

**Table 4:** Absorbed dose rate, annual effective dose rate, and total annual effective dose rate.

Site	Indoor dose rate (nGy/h)	Outdoor dose rate (nGy/h)	Indoor (AEDR) (mSv/y)	Outdoor (AEDR) (mSv/y)	Total (AEDR) (mSv/y)
Diani-1	202 ± 10	144 ± 7	0.61 ± 0.03	0.22 ± 0.01	0.83 ± 0.04
Diani-2	221 ± 11	160 ± 8	0.67 ± 0.03	0.24 ± 0.01	0.91 ± 0.04
Diani-3	248 ± 12	180 ± 9	0.70 ± 0.03	0.28 ± 0.01	0.94 ± 0.04
Diani-4	183 ± 9	130 ± 6	0.56 ± 0.02	0.20 ± 0.01	0.76 ± 0.03
Diani-5	292 ± 14	210 ± 10	0.89 ± 0.04	0.32 ± 0.02	1.21 ± 0.06
Tiwi-1	253 ± 12	183 ± 9	0.77 ± 0.03	0.28 ± 0.01	1.05 ± 0.05
Tiwi-2	204 ± 10	148 ± 7	0.62 ± 0.03	0.23 ± 0.01	0.85 ± 0.04
Tiwi-3	255 ± 12	184 ± 9	0.78 ± 0.03	0.28 ± 0.01	1.06 ± 0.05
Tiwi-4	265 ± 13	192 ± 9	0.81 ± 0.04	0.29 ± 0.01	1.10 ± 0.05
Tiwi-5	183 ± 9	131 ± 6	0.56 ± 0.02	0.20 ± 0.01	0.76 ± 0.03
Shelly-1	216 ± 10	157 ± 7	0.66 ± 0.03	0.24 ± 0.01	0.90 ± 0.04
Shelly-2	201 ± 10	146 ± 7	0.61 ± 0.03	0.22 ± 0.01	0.83 ± 0.04
Shelly-3	175 ± 8	127 ± 6	0.53 ± 0.02	0.19 ± 0.01	0.72 ± 0.03
Shelly-4	237 ± 11	171 ± 8	0.72 ± 0.03	0.26 ± 0.01	0.98 ± 0.04
Shelly-5	220 ± 11	160 ± 8	0.67 ± 0.03	0.25 ± 0.01	0.92 ± 0.04
Nyali-1	206 ± 10	151 ± 7	0.63 ± 0.03	0.23 ± 0.01	0.86 ± 0.04
Nyali-2	171 ± 8	124 ± 6	0.52 ± 0.02	0.19 ± 0.01	0.71 ± 0.03
Nyali-3	215 ± 10	156 ± 7	0.65 ± 0.03	0.24 ± 0.01	0.89 ± 0.04
Nyali-4	183 ± 9	134 ± 6	0.56 ± 0.02	0.21 ± 0.01	0.77 ± 0.03
Nyali-5	183 ± 9	133 ± 6	0.56 ± 0.02	0.20 ± 0.01	0.76 ± 0.03
Kenyatta-1	209 ± 10	150 ± 7	0.64 ± 0.03	0.23 ± 0.01	0.87 ± 0.04
Kenyatta-2	216 ± 10	156 ± 7	0.66 ± 0.03	0.24 ± 0.01	0.90 ± 0.04
Kenyatta-3	238 ± 11	173 ± 8	0.72 ± 0.03	0.27 ± 0.01	0.99 ± 0.04
Kenyatta-4	186 ± 9	135 ± 6	0.57 ± 0.02	0.21 ± 0.01	0.78 ± 0.03
Kenyatta-5	152 ± 7	110 ± 5	0.46 ± 0.02	0.17 ± 0.007	0.63 ± 0.03
Shanzu-1	219 ± 10	160 ± 8	0.67 ± 0.03	0.25 ± 0.01	0.92 ± 0.04
Shanzu-2	143 ± 7	102 ± 5	0.43 ± 0.02	0.16 ± 0.008	0.59 ± 0.02
Shanzu-3	180 ± 9	131 ± 6	0.55 ± 0.02	0.20 ± 0.01	0.75 ± 0.03
Shanzu4	198 ± 9	144 ± 7	0.60 ± 0.03	0.22 ± 0.01	0.82 ± 0.04
Shanzu-5	150 ± 7	108 ± 5	0.45 ± 0.02	0.17 ± 0.01	0.62 ± 0.03
Average	207 ± 8	150 ± 7	0.63 ± 0.03	0.23 ± 0.01	0.86 ± 0.04
World average [16]	84	54	0.41	0.07	

**Table 5:** Comparison of the average activity concentrations of^232^Th,^238^U, and^40^K in sands on selected Kenyan coastline beaches and other regions around the world.

Beach	Country	Ra_eq_ (Bq/kg)	*H*_in_	*H*_ex_	*E*_total_ (mSv/y)	References
Kenyan coast	Kenya	327	0.98	0.72	0.86	Present study
Lake Victoria shoreline	Kenya	367	1.17	0.99		Okelo et al., 2015
Tripoli	Libya	—	—	—	0.0054	El-Kameesy., 2008
Oniru Beach	Nigeria	179.1			0.1	
Nile delta (Rosetta beach)	Egypt	404.8		1.1		Mubarak et al., 2017
Al-arish	Egypt	229	—	—	0.14	Seddeek et al., 2004
Tamil Nadu	India	673.92	1.82	1.37	1.531	Suresh et al., 2014
Sithonia Peninsula	Greece				0.013 to 0.688	Papadopoulos et al., 2014

## Data Availability

The data used in this work will be made readily available whenever required.
